# Components of quality of life in hemodialysis patients from family caregivers’ perspective: a qualitative study

**DOI:** 10.1186/s12882-021-02584-8

**Published:** 2021-11-13

**Authors:** Sima Sadat Hejazi, Meimanat Hosseini, Abbas Ebadi, Hamid Alavi Majd

**Affiliations:** 1 Ph.D. Student in Nursing, Student Research Committee, Department of Community Health Nursing, School of Nursing and Midwifery, Shahid Beheshti University of Medical Sciences, Tehran, Iran; 2 Department of Community Health Nursing, School of Nursing and Midwifery, Shahid Beheshti University of Medical Sciences, Vali Asr Ave., Niayesh Cross Road, Niayesh Complex, Tehran, Iran; 3 Behavioral Sciences Research Center, Life Style Institute, Faculty of Nursing, Baqiyatallah University of Medical Sciences, Tehran, Iran; 4 Department of Biostatistics, Faculty of Paramedicine, Shahid Beheshti University of Medical Sciences, Tehran, Iran

**Keywords:** Caregivers, End-stage kidney disease, Hemodialysis, Quality of life

## Abstract

**Background:**

Patients with end-stage kidney disease experience serious complications which affect their lives. Few studies have investigated the patients’ quality of life qualitatively from the perspective of family caregivers as the closest individuals to the patients. The family caregivers are directly involved in the patients’ disease progression and observe the changes, problems, and complications of disease and hemodialysis. This study aimed to explain the components of quality of life in hemodialysis patients from the family caregivers’ perspective.

**Methods:**

In this qualitative inductive content analysis, 16 family caregivers of hemodialysis patients, presenting to the teaching hospitals of Tehran, Iran, were enrolled via maximum-variation purposive sampling; sampling continued until reaching data saturation. The data collection method included in-depth semi-structured interviews. Also, an inductive content analysis was carried out based on Elo and Kyngas’ method.

**Results:**

A total of 311 codes, 19 subcategories, eight generic categories, and three main categories were extracted in this study. The main (and the generic categories) included mental and psychological problems (depressive mood, incompatibility and reduced tolerance, mental exhaustion, and deprivation of basic needs), social disruption (social isolation and social threats), and physical problems (general complications and disabilities and defects in the normal functioning of organs).

**Conclusion:**

Family caregivers can be valuable information sources for formal caregivers to plan treatment for chronically ill patients who are mainly cared for at home. The present results can help us increase the existing knowledge on the impact of end-stage kidney disease and hemodialysis on the patients’ quality of life. It seems that addressing the issues related to quality of life, mentioned by the caregivers, can positively affect the patients’ quality of life and even reduce the caregivers’ burden.

## Background

End-stage kidney disease (ESKD) is a life-threatening condition in which the kidneys lose their normal function [1]. Patients with this disease experience serious complications, which affect their lives [2] and compromise their physical, mental, and emotional health [1]. Physiological stressors that cause limitations for the patients can disrupt their daily lives. These complications include diet and fluid restrictions, decreased mobility, medication use, treatment-related complications, vascular access problems, and long-term treatment. Besides, the pathological nature of ESKD is associated with fluid retention, hypertension, renal osteodystrophy, cardiovascular disease, and sexual dysfunction. Psychological symptoms are also common in these patients, including fatigue, irritation, depression, anxiety, isolation, and work-related problems [3].

Patients with ESKD need renal replacement therapies to survive [1]. Previous studies have reported a low quality of life (QoL) in hemodialysis patients [4]. Both hemodialysis and kidney transplantation reduce the patients’ health-related QoL (HRQoL) [4, 5]. Nevertheless, according to previous studies, transplant patients have a higher HRQoL compared to hemodialysis patients [5]. Various studies on different populations have reported the negative impact of chronic kidney disease and hemodialysis on the patients’ QoL, functional status, and personal and social relationships [1]. The chronic nature of the disease and treatment constitute long-term stressors that negatively affect the patient’s health and daily life [3]. Overall, the limitations of hemodialysis affect all aspects of a patient’s life, including physical performance, sexual activity, employment, and financial status [6].

The World Health Organization (WHO) has defined QoL as “an individual’s perception of their position in life in the context of the culture and value systems in which they live and in relation to their goals, expectations, standards, and concerns. It is a wide-ranging concept, influenced in a complex way by a person’s physical health, psychological state, level of independence, social relations, and relationship with the salient features of their environment.” [7]. Besides, HRQoL assesses the effect of illness and therapy on the well-being of different individuals and is related to the physical, emotional, mental, and social status of the patients [8]. According to these definitions, the present study evaluated the impact of hemodialysis on the patients’ physical health, psychological state, and social relations.

Previous studies have reported a low QoL in hemodialysis patients [4]. Informal caregivers play a fundamental role in treatment by providing practical and emotional support for the patients and facilitating treatment follow-ups [9]. Overall, family caregivers play a vital role in caring for disabled family members or friends [10], and families are recognized as the most important sources of support and care for chronically ill patients [11]. In addition to the patient, dialysis treatment affects the entire family [12]. The family caregiver is responsible for managing different problems of ESKD patients [13]. Numerous studies have evaluated the QoL of hemodialysis patients from the patients’ point of view or have examined patient-reported outcome measures [14–18]; however, most of these studies are quantitative [6].

The review of the literature indicated that few studies have investigated the patients’ QoL qualitatively from the perspective of the family caregiver as the closest individual to the patient, who is directly involved in the disease process and observes the changes, problems, and complications associated with ESKD and hemodialysis. To address the existing knowledge gap, the present study aimed to qualitatively explain the components of QoL in hemodialysis patients from the family caregivers’ perspective.

## Methods

### Study design

The inductive content analysis method [19] was applied in this qualitative study.

### Sampling method

The participants included the family members or relatives of hemodialysis patients, who were responsible for providing informal care. They were selected through a purposive sampling method with maximum variation in terms of age, gender, occupation, education, and relation to the patient. In qualitative research and purposeful sampling, researchers seek to find people with extensive experience in the research concept (key informants). In this study, the participants were identified by the researcher at the hospitals as the research setting. One of the researchers communicated with the patient and the caregiver in the hemodialysis ward. After introducing herself and the research objectives, the caregivers were invited to participate in the study. The study setting included the teaching hospitals of Tehran, Iran.

The inclusion criteria were as follows: willingness to participate in the study; caring for a patient over the age of 18 years; being the patient’s main caregiver; speaking Persian; and not being paid for caregiving services. To achieve maximum variation in sampling, the researcher sampled individuals presenting to hospitals in different parts of the city. There was no predetermined sample size, and sampling continued until reaching data saturation.

### Data collection

The data collection method included in-depth, semi-structured, face-to-face interviews with open-ended questions. The first author, trained in qualitative research, conducted the interviews from June to September 2020. The interview guide was developed and examined in a sample. It contained open-ended questions based on the study objectives, which were formulated after consulting the research team. Some guide questions were asked in the interviews (e.g., “What problems did your patient have after starting hemodialysis?” and “In your experience, when can you say that a patient has a high quality of life considering his/her conditions?”). Moreover, based on the data flow, exploratory questions were asked from the participants to deepen our understanding of their experiences. The interview guide is presented in Table 1.

**Table 1:** The interview guide

**Introductory questions**	
How long has your patient been under hemodialysis? Can you tell me if you are living with your patient in the same household? What is your relation to the patient?	
**Patient’s quality of life from the caregiver’s perspective**	
Can you describe one day of your patient’s life with and without dialysis from morning till night? What problems did your patient experience after starting hemodialysis? Can you explain how dialysis has affected your patient’s life? In your experience, when can you say that a patient has a high quality of life considering their condition?	
**Demographics**	
What is your educational level? What is your marital status? How old are you?	
**Closing questions**	
Before the end of the interview, do you have anything else to say about the patient, her/his condition, or quality of life? Do you have any questions?	

The time of the interviews were arranged according to the participants’ desires, and the interviews were held at in the companion’s waiting room in the hemodialysis wards of teaching hospitals in Tehran, Iran, in the absence of any outsiders. Before the interviews, written informed consent was obtained from the participants. The interviews were recorded with the participants’ consent. The demographic characteristics were also recorded in relevant forms. No interviews were repeated, and no participants withdrew from the study. Finally, 16 family caregivers of hemodialysis patients were interviewed. The average duration of the interviews was 35.55 min.

### Data analysis

Data analysis was performed simultaneously with data collection. The interviews were recorded and transcribed verbatim by the first author. Data were analyzed using an inductive content analysis approach, based on Elo and Kyngas’ method [19], which consisted of three phases, namely, preparation, organization, and reporting the process of data analysis and reaching the results. MAXQDA v.18 was also used for the management of data. The interviews were conducted after selecting the data analysis unit (i.e., interview transcripts), determining the sampling method, developing the interview guide, and deciding on the analysis of both explicit and implicit contents in the preparation phase. Next, open coding was performed in the organization phase, and a codebook was developed.

In this study, consensus coding was employed. The first and second authors coded the interviews. The coding results were then reviewed in several sessions by the research team, and a single, agreed-upon coding was achieved. Next, data abstraction was performed by forming subcategories, generic categories, and main categories. In the final phase, the analysis process and the findings were reported. The combination of the following criteria was used to assess data saturation: achieving high-prevalence codes; assessing new data after a new interview and the number of participants interviewed (more than nine participants); and appraisal of achieving meaning saturation and extensive understanding of the research concept (based on consultation with the research team) [20, 21].

### Data accuracy and rigor

Guba and Lincoln (1994) proposed credibility, dependability, transferability, and confirmability to describe practical techniques for achieving data rigor [22]. This study used these criteria to measure the rigor of data. Peer and member checking was carried out to assess the credibility of data; from the peers’ perspective, none of the items required revision or correction. Besides, to achieve dependability, full explanations were given to the participants about the study before the interviews, and identical questions were asked. Also, documents related to the data analysis process were registered for evaluation by an external auditor. Quotations were used to report the results. Moreover, to achieve transferability, attempts were made to present a clear report of the findings. Also, to ensure confirmability, the participants were asked to provide further explanations if their response was ambiguous.

## Results

Sixteen family caregivers were included in this study, none of whom were eliminated. Twelve participants were female, and four were male. Other demographic characteristics of the participants are presented in Table 2. Overall, 311 codes, 19 subcategories, eight generic categories, and three main categories were extracted, as shown in Table 3.

**Table 2:** The demographic characteristics of the participants

Number	Educational level	Relationship to the patient
1	Bachelor’s degree	Daughter
2	PhD	Daughter
3	High school diploma	Son
4	High school diploma	Wife
5	Master of Science	Son
6	High school diploma	Wife
7	High school diploma	Husband
8	Bachelor’s degree	Wife
9	High school diploma	Wife
10	Illiterate	Wife
11	Primary school	Wife
12	Illiterate	Wife
13	Bachelor’s degree	Son
14	Bachelor’s degree	Daughter
15	High school diploma	Wife
16	Bachelor’s degree	Daughter

**Table 3:** The process of abstraction of subcategories, generic categories, and main categories

Main categories	Generic categories	Subcategories
Mental and psychological problems	Depressive mood	Low spirits
		Despair and wish for death
		Mental conflict and impatience
	Incompatibility and reduced tolerance	Ignoring the disease and treatment
		Nervousness, inflexibility, and irritability
		Emergence of abnormal behaviors
	Mental exhaustion	Fear and stress
		Suffering
		Exhaustion and tension
	Deprivation of basic needs	Sexual dysfunction
		Sleep disorders
Social disruption	Social isolation	Feelings of loneliness and isolation
		Dependence on the caregiver
	Social threats	Employment threats
		Stigmas and social labels
Physical problems	General complications and disabilities	General physical complications
		Gradual weakness and disability
	Defects in the normal functioning of organs	Mobility and musculoskeletal disorders
		Neurological disorders

### Main category 1: mental and psychological problems

This category consisted of generic subcategories, including depressive mood, incompatibility and reduced tolerance, mental exhaustion, and deprivation of natural needs, which are described below:

#### Depressive mood

The family caregivers of hemodialysis patients believed that their patients experienced some psychological problems. Some of them believed that their patients were low-spirited and had symptoms of depression. In this regard, one of the participants said:

*“My mother often cries.”* (P3, the patient’s son)

Also, another participant stated:

*“She is depressed now; she is so down.”* (P7, the patient’s husband)

One of the issues discussed by the caregivers was the patient’s despair. From their point of view, the patients felt that their life was over; in other words, they were waiting for their death and were not hopeful about the future. In this regard, one of the participants said:

*“You see, he has no hope for a good future. He feels that there is nothing left of his life.”* (P4, the patient’s wife)

Moreover, participant No. 2 said:

*“They are all sad and depressed; they are hopeless. They ask if they can die sooner or wish for death. They are very upset and give up on life.”* (P2, the patient’s daughter)

According to the caregivers, hemodialysis had become a constant preoccupation for the patients. Besides, the patients gradually grew impatient and constantly thought about hemodialysis:

*“She is lost in her thoughts of hemodialysis. If she has dialysis today, she will think about it all day; she will also think about it the day after. She gets really annoyed by it, which is quite noticeable. It’s completely tangible.”* (P5, the patient’s son)

The constant mental conflicts of patients cause communication problems, depression, and in many cases, sleep disorders and anxiety. In this regard, one of the caregivers said:

*“ … My father isn’t in a good mood when he comes back from dialysis and goes right back to his bed; he has no patience for doing anything.”* (P14, the patient’s daughter)

#### Incompatibility and reduced tolerance

Some caregivers stated that their patients had not yet accepted hemodialysis as an inevitable procedure during their life. They argued that the patients ignored the limitations of the disease and hemodialysis and considered them to be transient and terminable:

*“My mother doesn’t still believe that hemodialysis needs to continue until the end of her life.”* (P3, the patient’s son)

Also, participant No. 5 stated:

*“One of our problems is that she hasn’t yet accepted that she has this disease and that she must deal with it. Because she doesn’t accept it, she gets more annoyed by it.”*

The caregivers stated that their patients became more nervous after starting hemodialysis. In this regard, one of the participants said:

*“Honestly, she’s become a little bit stressed, nervous, stubborn, and sensitive about some issues; it has become worse since dialysis.”* (P13, the patient’s son)

Meanwhile, some caregivers noticed that the patients stubbornly resisted the caregivers’ advice and reacted harshly if anything was undesirable to them. One of the participants remarked:

*“He’s become like a little kid. He’s spoiled and stubborn and reacts harshly when I say anything slightly undesirable to him as if I was cursing or beating him up.”* (P12, the patient’s wife)

The caregivers also reported that their patients showed new abnormal behaviors after starting hemodialysis:

*“…He pretends to be oppressed by me. He’s become very greedy for money. I mean if the kids ask him if he needs money, he does not say no, although we have everything we need in our house, and we also have money … ”* (P4, the patient’s wife)

According to some caregivers, the patients believed that the caregiver was cruel or uncompassionate toward them or would intentionally harass them. In this regard, a caregiver said:

*“He thinks I'm oppressing him.”* (P6*,* the patient’s wife)

#### Mental exhaustion

From the caregivers’ perspective, fear and stress were other psychological problems of hemodialysis patients. Generally, the patients become stressed after the diagnosis and the onset of hemodialysis. One of the participants said:

*“She’s nervous and stressed.”* (P3, the patient’s son)

Moreover, participant No. 5 made the following remark about her mother:

*“She gets so stressed the night before hemodialysis that she can't sleep.”* (P5, the patient’s son)

Suffering is one of the psychological issues affecting hemodialysis patients. The caregivers believed that hemodialysis and its requirements, besides limitations imposed by ESKD, cause suffering for the patients. One of the participants believed that painful catheter insertion, which is repeated 3 days a week, causes suffering for the patient:

*“Well, these catheter insertions bother her.”* (P13, the patient’s son)

From the perspective of some caregivers, the food and fluid intake restrictions in the patients’ therapeutic regimen are associated with suffering. Participant No. 10 said:

*“Because my husband is a food lover and is always thinking about eating, it is very challenging for him to avoid eating most things. It’s so difficult.”* (P10, the patient’s wife)

The majority of the caregivers stated that exhaustion was one of the persistent problems of their patients. In this regard, participant No. 4 said:

*“It is as if he is exhausted all the time. He doesn’t want to commit to anything; he says he is exhausted or bored.”* (P4, the patient’s wife)

Participant No. 2 also stated:

*“They get so exhausted and bored under the machine.”* (P2, the patient’s daughter)

#### Deprivation of basic needs

The nature of ESKD and the psychosocial consequences of this disease and its treatment cause sexual dysfunctions in hemodialysis patients. In this study, the caregivers who were the patients’ spouse reported sexual dysfunction in their partners. Participant No. 8 said:

*“He has sexual desires, but he can’t perform … He should take pills.”* (P8, the patient’s wife)

Moreover, participant No. 15 said:

*“Their sexual desire is majorly reduced. Even if they have it, they can’t reach an orgasm. All patients are the same in this area. I’ve asked around.”* (P15, the patient’s wife)

These problems can lead to some emotional and communication problems between the patients and their caregivers (spouses) and create a feeling of guilt in the patients. Besides, the caregivers of hemodialysis patients witnessed sleep disorders in the patients. Some of them reported oversleeping in the patients, while some reported sleep disturbances or short, disturbed episodes:

*“He sleeps all the time … For example, we had no dialysis session yesterday, and I was at home from 7:30 in the morning until 7:00 in the evening. He got up, had breakfast, and slept again.”* (P14, the patient’s daughter)

Also, participant No. 7 said:

*“She sleeps in short, disturbed episodes.”* (P7, the patient’s husband)

### Main category 2: social disruption

The category of social disruption consisted of two generic categories of social isolation and social threats, which are described below:

#### Social isolation

The caregivers of hemodialysis patients witnessed the negative impact of ESKD and hemodialysis on the patients’ social life. They stated that their patients were reluctant to socialize after the onset of their disease and treatment and expressed their feeling of loneliness:

*“Since she has had the permacath, she doesn’t like to join social gatherings. She’s told us not to let anyone come and visit her. She doesn’t like it.”* (P2, the patient’s daughter)

Participant No. 13 said:

*“She’s become a little too reserved to express herself or vent her feelings.”*

Also, participant No. 5 said:

*“She feels lonely; this disease has made it worse.”* (P5, the patient’s son)

One of the other problems of the caregivers was the increased dependence of their patients on them. Participant No. 7 said:

*“She’s anxious and restless about always having me or someone else by her side. Her dependence on me has grown.”* (P7, the patient’s husband)

#### Social threats

According to the caregivers, many hemodialysis patients lose their job due to fatigue, weakness, or need for hemodialysis, leading to social problems and isolation:

*“He can’t go to work anymore; now, he’s at home all the time and doesn’t work … ”* (P11, the patient’s wife)

According to the caregivers, the patient’s job loss, especially when it is the only source of family income, imposes a financial burden on the family. This burden negatively affects the QoL of patients and their families.

The caregivers stated that some of the patients’ relatives behaved as if the patient had a contagious disease:

*“You know, some relatives think he’s infected.”* (P4, the patient’s wife)

This social stigma constitutes a social problem for the patient. Also, according to some caregivers, changes in the patients’ appearance due to the disease trigger negative reactions in people in the community, causing the patients to limit their social interactions.

#### Main category 3: physical problems

This main category consisted of two generic categories, namely, general complications and disabilities and defects in the normal functioning of organs.

#### General complications and disabilities

The caregivers reported multiple symptoms and physical problems in the patients. They described numerous general problems, such as loss of appetite, nausea, and fluctuations in blood pressure, which were the main complaints of the patients. One of the caregivers stated:

*“She has little appetite. She eats a tiny amount of bread (she shows the size of bread with her fingers) for breakfast.”* (P2, the patient’s daughter)

Moreover, two other participants stated:

*“Her calcium level has dropped due to the disease, all of her teeth have fallen out, and now, we have to remove all of her teeth.”* (P3, the patient’s son)

*“They develop hypotension during hemodialysis, which is very annoying for them.”* (P6*,* the patient’s wife)

According to the caregivers, hypotension led to weakness, activity intolerance, and dependence on the caregivers for the activities of daily living. Also, it increased the risk of falling in the patients. The caregivers believed that dealing with weakness was an inevitable part of the patients’ lives after the onset of the disease and hemodialysis. They had witnessed a gradual decline in the patients’ abilities:

*“The problem is that they don’t feel well after hemodialysis. He’s not ok at all after it. He only rests afterward until it is time to come back again.”* (P11, the patient’s wife)

*“He’s always in bed.”* (P6*,* the patient’s wife)

*“Her ability is deteriorating day by day…”* (P5, the patient’s son)

Therefore, hemodialysis and the nature of ESKD affect the physical health and well-being of patients and decrease their QoL.

#### Defects in the normal functioning of organs

Musculoskeletal and mobility disorders were among the caregivers’ problems while caring for the patients. These disorders included muscle cramps, increased susceptibility to bone fractures, experience of bone fractures with minor traumas, and mobility difficulties. One of the participants said:

*“He gets muscle cramps after dialysis.”* (P15, the patient’s wife)

Also, according to the caregivers, imbalance, dizziness, and delirium were some other problems of hemodialysis patients. One of the participants said:

*“She may fall; she may lose her balance; she doesn’t feel steady.”* (P1, the patient’s daughter)

Also, participant No. 5 said:

*“My mother is delirious. Her delirium stems from continuous dialysis sessions.”*

## Discussion

This study aimed to identify the components of QoL in hemodialysis patients from the perspective of their family caregivers. The caregivers believed that the patients’ QoL was affected by the disease physically, psychologically, and socially. The results of the present study are matched with the results of studies conducted on the patients themselves. In Asian countries, including Iran, the strong family structure and bonds, cultural norms, including filial obligations, community expectations, and social factors and a sense of closeness between family members may lead to similarities in the perceptions of caregivers and hemodialysis patients about QoL.

Regarding the physical aspect, Han et al. reported physical problems, such as nausea, anorexia, and weakness [14], which is consistent with the present study. Moreover, in a thematic synthesis [23], a limited life was one of the extracted themes, emphasizing the physical and mental limitations of these patients. Moreover, in most qualitative studies reviewed in a meta-analysis by Bayhakki and Hatthakit, fatigue was discussed as one of the physical limitations. Fatigue and weakness were also reported as common physical problems after dialysis [24].

Additionally, a review of qualitative studies by Roberti et al. [25] showed that the physical and mental capacity of hemodialysis patients for managing their daily life was negatively affected and limited by the symptoms of their disease and hemodialysis. Dietary and fluid restrictions, the nature of hemodialysis treatment, toxin accumulation and uremic syndrome, fluid overload, and metabolic disorders contributed to weakness, fatigue, and lack of energy in hemodialysis patients [24] and were recognized as common physical complications in these patients; these physical problems generally affect the well-being and QoL of the patients.

Skin problems and itching were among other physical problems of hemodialysis patients in various studies [14, 26]; however, the caregivers did not report any specific complications in the present study. The reason for this disparity in the findings might be that in the present study, problems were discussed from the caregivers’ perspective, whereas in the mentioned study, the patients’ perspective was investigated. Besides, some other complications were probably more significant from the caregivers’ perspective.

In the present study, the participants emphasized on sleep problems of a psychological origin, although sleep problems of a physical origin were also mentioned. Nonetheless, sleep problems were classified in the psychological category, given the higher frequency of psychological factors. On the other hand, in a study by Han et al. [14], sleep disorders, which were one of the complications of hemodialysis patients, were categorized as physical problems. Moreover, in a quantitative study by Parvan et al. [27], 83% of hemodialysis patients had a poor sleep quality, and there was a significant negative correlation between sleep quality and QoL. Anxiety and stress (especially the night before dialysis), along with weakness, muscle pain, and cramps, can change the sleep pattern. The result of a study on diabetic patients [28] showed that sleep disorders were related to a poor HRQoL.

The patients’ low spirits and depression were mentioned by the caregivers in the present study. The results of various studies have indicated the higher prevalence of depression in patients with chronic kidney disease [29]. Patients with ESKD are five times more likely to develop depression compared to normal individuals [30]. In Roberti’s review study, depression was identified as a factor reducing the patients’ physical and mental abilities [25]. Moreover, the results of Finnegan-John and Thomas indicated depression as a recurrent response to loss and stress caused by the disease [16]. The results of these studies are consistent with the caregivers’ statements in the present study. Overall, physical and social limitations of hemodialysis patients, along with stress, anxiety, fear of the future, dependence on others, and dialysis treatment, make depression inevitable. According to the results of previous studies, depressed patients have an impaired QoL [31–33].

One of the issues raised by the caregivers in this study was threats to the patients’ employment status. The review study by Roberti et al. [25] similarly showed that the patients who were physically capable of maintaining their job commonly had informal or temporary jobs with lower income; other patients were inevitably unemployed. Overall, physical weakness, fatigue, reduced activity capacity, time spent on hemodialysis, and follow-up of various medical problems and psychiatric disorders, such as depression, pose risks to the patient’s employment status, which can exacerbate their psychological symptoms and increase the financial and social pressures on them. On the other hand, social and financial pressures may lead to a low QoL in these patients and impose a significant caregiving burden on the caregivers.

Thoughts about death and wish for death were one of the subcategories extracted from the interviews in this study. In this regard, Finnegan-John and Thomas similarly reported thoughts of death in many interviewees [16]. They believed that a background of depression, along with frustration, future uncertainties, financial burdens, limited social relations, and feeling of isolation, may lead to thoughts of death in many patients. On the other hand, depression and death-related thoughts cause isolation. Isolation, unwillingness to communicate with others, and constant mental preoccupation with negative thoughts cause the patients to experience psychological problems, which in turn reduce their QoL.

Moreover, reduced sexual desire and potency were among other subcategories extracted in the present study. Similarly, hemodialysis patients examined in studies reviewed by Roberti et al. reported sexual problems [25]. Another study showed that sexual function and intimate relationships of the patients were negatively affected [16]. Overall, the chronic nature of kidney disease and the subsequent hormonal disorders, stress, depression, and financial and social pressures can lead to sexual dysfunctions in hemodialysis patients. Concerns about sexual relationships can affect the patient’s QoL. The interconnectedness of the patients’ psychological issues, besides the impact of psychological problems, such as depression and anxiety, on different aspects of life, highlights the need for increased attention to psychological issues in these patients. Previous studies have reported the negative effect of psychiatric disorders on therapeutic outcomes in patients with a kidney disease. Overall, depression in a patient with chronic kidney disease is associated with a high risk of non-adherence, increased hospitalization and mortality, high expenditure, and need for specialists [29].

In the present study, the caregivers reported various abnormal behaviors in their patients. Similarly, Rezaei et al. showed a decrease in the tolerance threshold, increased irritability, poor anger management, impatience, and behavioral changes in patients undergoing hemodialysis [34]. Various physical, psychological, and social pressures, feelings of loneliness and isolation, lack of supportive resources, and dependence on dialysis may lead to uncontrolled and aggressive behaviors in hemodialysis patients and affect their QoL. On the other hand, these inappropriate behaviors negatively affect the patient-family relationship and the family caregiver and increase the burden of care.

In the present study, the caregivers reported their patients’ reluctance to communicate with others and mentioned their tendency toward isolation. One of the themes extracted by Lee et al. was intentional isolation. The participants sought isolation because of their lack of interest and motivation in life and indifference to their environment [35].

The present study focused on the caregivers of hemodialysis patients in Tehran, the capital of Iran. Although qualitative studies, such as the current study, do not consider generalizing the findings, the inclusion of caregivers from different ethnic groups and cities of Iran could have increased the richness of our findings; however, it was not possible in this study.

## Conclusion

In this study, the viewpoints of family caregivers about different aspects of hemodialysis patients’ QoL (physical, psychological, and social dimensions) were investigated. The present results can help us bridge the existing knowledge gap regarding the impact of ESKD and hemodialysis on the patients’ QoL. Caregivers can be valuable information resources for formal caregivers to plan care for chronically ill patients, who are mainly cared for at home. Overall, attention to different aspects of QoL, mentioned by the caregivers, and addressing the related issues can positively affect the patient’s QoL and even reduce the caregiver’s burden. Future studies are recommended to evaluate the effectiveness of different measures to improve the QoL of hemodialysis patients not only from the patients’ perspective, but also from their caregivers’ point of view.

## Data Availability

The datasets used and analyzed during the current study are not publicly available due to the sensitive nature of the qualitative questions asked in this study but are available from the corresponding author on reasonable request.

## Abbreviations

ESKD: End-Stage Kidney Disease
QoL: Quality of Life
HRQoL: Health-Related Quality of Life
WHO: World Health Organization
